# Serum 8-isoprostane levels in patients with resistant oral lichen planus before and after treatment with lycopene: a randomized clinical trial

**DOI:** 10.1186/s12903-021-01711-z

**Published:** 2021-07-15

**Authors:** Aliaa Abdelmoniem Bedeir Eita, Azza Mohamed Zaki, Sabah Abdelhady Mahmoud

**Affiliations:** 1 Oral Medicine, Periodontology, Diagnosis and Radiology Department, Faculty of Dentistry, Alexandria University, Alexandria, Egypt; 2 Medical Biochemistry Department, Faculty of Medicine, Alexandria University, Alexandria, Egypt

**Keywords:** Isoprostanes, Lycopene, Oral lichen planus, Oxidative stress, Prednisolone

## Abstract

**Background:**

Oral lichen planus is an autoimmune disease in which topical steroids are the first line of treatment. The adverse effects of systemic corticosteroids prescribed for resistant oral lichen planus cases advocate alternative modalities. Lycopene is an antioxidant with a wide range of beneficial properties. This trial aimed to evaluate the effect of pure lycopene as compared to systemic corticosteroids (Prednisolone) on the symptoms, signs and oxidative stress in patients with erosive oral lichen planus recalcitrant to topical steroids.

**Methods:**

Twenty patients were randomly divided into the test (lycopene) and control (corticosteroids) groups. Numeric rating scale and Escudier et al. (Br J Dermatol 4:765–770, 2007. 10.1111/j.1365-2133.2007.08106.x) lesion scores were assessed at baseline and weeks 4 and 8 from baseline. Serum levels of 8-isoprostane were measured in all patients at baseline and at the end of treatment (week 8).

**Results:**

There was a significant reduction in signs and symptoms after the end of treatment in each group. However, no significant difference was found between the lycopene and the corticosteroids group. Moreover, a significant reduction in 8-isoprostane levels was observed in the lycopene group from baseline and as compared to the control group.

**Conclusions:**

Based on the study results, lycopene is a safe and effective therapeutic modality for resistant oral lichen planus. 8-isoprostane is a biomarker of lipid peroxidation that can be reduced by lycopene.

*Trial registration ID*: PACTR202003484099670. 'Retrospectively registered on 11/3/2020'.

**Supplementary Information:**

The online version contains supplementary material available at 10.1186/s12903-021-01711-z.

## Background

Lichen planus (LP) is an inflammatory disease of autoimmune nature [1]. Oral lichen planus (OLP) is a pan racial disorder occurring in about 0.55–2% of the population [2, 3] with diverse patterns of oral lesions. Reticular, papular, plaque, atrophic, bullous, and erosive forms all function as oral variants that can sometimes show up in a patient at the same time with varying degrees of predominance [4].

To date, the nature of the OLP antigen is unclear. However, the disease is found to be initiated by apoptosis of basal keratinocytes, where auto-cytotoxic T lymphocytes (CD8+) are the triggering cells to such a process [5].

Reactive oxygen species (ROS) and free radicals are unstable molecules that, when released in numerous amounts, are capable of inducing inflammatory and immune responses [6, 7]. They hereby are said to put cells in a state of oxidative stress [8]. Oxidative damage to cellular DNA, proteins, and lipids is considered the outcome of oxidative stress giving rise to a wide array of diseases including OLP [9, 10].

8-isoprostane (8-iso-PGF2α) is a prostaglandin isomer of the F2 isoprostane family. It is released in different body fluids as a result of the oxidation of cellular membrane arachidonic acid and is considered a reliable and stable biomarker of lipid peroxidation and oxidative stress in various diseases and conditions including OLP [11, 12].

Topical corticosteroids are the treatment of choice for symptomatic OLP. In cases of extensive oral lesions or no response to topical therapy, systemic corticosteroids are indicated [13]. However, oral steroids can cause adverse side effects as fluid retention, hyperglycemia, peptic ulcer, increased susceptibility to infection, and others [14].

The effect of various antioxidants has been widely evaluated as defense systems against the free radical-mediated oxidation process in an attempt to discover a definitive safe therapeutic modality for OLP [4]. Lycopene (LYC) is the fat-soluble red carotenoid pigment found in fruits and vegetables [15]. Its health and disease benefits lie in being a strong quencher of free radicals and ROS mainly singlet oxygen. The biological properties of lycopene mark its role in various diseases, particularly oral mucosal diseases [16]. For all those reasons, along with considering the implication of oxidative stress in OLP pathology, lycopene is believed to exert significant effects in the treatment of OLP and the prevention of its malignant transformation [7].

This clinical trial aimed to evaluate the therapeutic effect of pure systemic lycopene as a single treatment for patients with EOLP who are unresponsive to topical corticosteroids in comparison to systemic Prednisolone according to the reduction in clinical signs and symptoms and to measure the serum level of 8-isoPGF2α in all patients before and after treatment with both therapeutic modalities.

## Methods

### Study design and ethical considerations

A Parallel randomized controlled clinical trial following the CONSORT guidelines [17] was conducted on 20 Patients with EOLP attending the outpatient clinic of the Oral Medicine, Periodontology, Diagnosis, and Radiology Department, Faculty of Dentistry, Alexandria University, Egypt. They were diagnosed according to the modified WHO criteria of oral lichen planus *2003* in terms of history, clinical and histopathological examination [18]. Enrollment was performed by the study operators. Patients were treated according to the principles of the modified Helsinki's code for human clinical studies *2013*.

Inclusion criteria involved male and female patients aged from 30 to 60 years who were previously treated by topical corticosteroids (0.1% Triamcinolone Acetonide gel) along with topical antifungal (2% Miconazole gel) three times daily for at least six consecutive weeks, they presented to the Oral Medicine clinic with only mildly improved yet felt pain and persistent oral lesions and are defined as unresponsive OLP patients to the conventional topical steroids therapy [19–21].

Exclusion criteria involved smoking and tobacco use in any form, pregnant and lactating females, patients with suspected lichenoid contact/drug reactions, patients with medical history and laboratory investigations that suggest the presence of systemic diseases (Diabetes, liver disease, renal disease and any other autoimmune or collagen disease), lesions showing any dysplastic changes in the biopsy specimen and patients having cutaneous LP.

Patients were randomly allocated into one of two groups namely lycopene (test) and corticosteroids (control) groups using the permuted block randomization technique, and the block size was 2 [22]. Allocation code was concealed from an uninvolved examiner in the study who allocated the participants to the intervention arms using sealed opaque envelopes [23]. Blinding was carried out by masking the type of intervention from the biochemist as an outcome assessor and statistician as a data analyst [24]. Blinding of the operators and patients was difficult due to the different doses, formulations, and modes of administration of therapeutic agents in both groups.

Sample size was estimated assuming alpha error = 5% and study power = 80%. Based on a pilot study conducted on 5 resistant patients with EOLP for 8 weeks, mean ± standard deviation (SD) numeric rating scale (NRS) = 1.90 ± 0.72 after lycopene administration, and = 3.12 ± 0.91 after corticosteroids administration. Based on comparison of means, a minimum sample size was calculated using G* Power software to be 9 per group which was increased to 10 assuming a dropout rate of 10% (effect size = 1.49) [25–27]. The total sample size included 20 patients.

### Interventions

- Participants of the lycopene (test) group were administered 10 mg of lycopene soft gel capsules as a single morning dose for eight consecutive weeks [7, 28]. The active ingredient in each capsule consists of 10 mg lycopene from natural tomato extract.
- Participants of the corticosteroids (control) group were administered 40 mg (2 tablets) of oral Prednisolone as a single morning dose for four consecutive weeks and the dose was tapered along another four weeks. Incremental reduction of 10 mg each week for the first three weeks, followed by 5 mg reduction in the last week, was the tapering protocol in this study [7, 13, 29]. Each tablet's active ingredient consists of Prednisolone metasulfobenzoate sodium 31.44 mg, which is equivalent to 20 mg of Prednisolone.
- In both study groups, calculus and all sources of traumatic irritation were removed. Also, all patients were instructed about the proper oral hygiene procedures [30]. Moreover, compliance was checked upon by phone calls to all patients weekly.
- Peripheral blood samples were collected from all enrolled patients before and after treatment. Blood was driven at the outpatient clinic of the Oral Medicine, Periodontology, Diagnosis, and Radiology Department, Faculty of Dentistry, Alexandria University, Egypt following the WHO guidelines on drawing blood *2010* [31]. Samples were sent in evacuated red-grey topped serum separator tubes to the laboratory of the Biochemistry Department at Faculty of Medicine, Alexandria University, Egypt, on the same day of its collection. Each tube was coded to its corresponding patient. An additional letter code differentiated baseline and post-treatment samples beside the original code. In the lab, samples were stored at room temperature for 2 h or put at 4 °C overnight and centrifuged for 20 min at approximately 1000×*g* to separate serum. Serum was aspirated using a pipette, aliquoted into Eppendorf tubes, and stored at − 20 °C until the analysis time [32].

### Primary outcome measure

Subjective assessment was conducted at baseline and after 4 and 8 weeks from baseline using the numeric rating scale (NRS) [33]. The NRS is represented as a plain horizontal 10 cm line. Patients were instructed to bisect the line at a point appropriate to their present discomfort. A zero value equates to being pain-free, whereas the most severe pain they have experienced was rated at 10.

### Secondary outcome measures

Biochemical assessment was conducted by measuring serum 8-isoprostane levels at baseline and eight weeks from baseline using Human 8-iso-PGFα (8-isoprostane) ELISA kit purchased from Biomatik, Cambridge, Ontario**,** Canada. A biochemist prepared the reagents and performed the assay procedure at the Faculty of Medicine's Biochemistry lab following the manufacturer's instructions. Quantitative analysis of 8-isoprostane in Picograms per milliliters (pg/ml**)** was based on setting a standard curve by plotting an average optical density (OD) of 450 nm for each standard solution concentration on the vertical (Y) axis versus its corresponding concentration of testing sample on the horizontal (X) axis**.** To determine the amount in each sample, the OD value was located on the Y-axis. At the point of intersection between a horizontal line drawn from each value and the standard curve, the corresponding concentration of each testing sample was obtained on the x-axis along a vertical line drawn from that point.

Objective assessment of oral lesions was conducted at baseline and after 4 and 8 weeks from baseline using the criteria set by Escudier et al. [34]. Seventeen oral sites were examined for evidence of OLP in every patient. Site and severity scores were taken as follows: Site score: 0, no detectable lesion present; 1, evidence of lichen planus seen; 2, > 50% of buccal mucosa, dorsum of tongue, floor of mouth, hard palate, soft palate or oropharynx affected. Severity score: 0, keratosis only; 1, keratosis with mild erythema (< 3 mm from gingival margins); 2, marked erythema (e.g. full thickness of gingivae, extensive with atrophy or edema on nonkeratinized mucosa); 3, ulceration present. An activity score was taken by multiplying the values of site and severity scores of each involved site.

### Statistical analysis

Normality was checked for all variables using descriptive statistics, plots, and normality tests. Means and standard deviations (SD) were calculated for normally distributed variables (pain using numeric rating scale), in addition to non-normally distributed variables (lesion scores and 8-isoprostane levels). For non-normally distributed variables, median and Inter Quartile Range (IQR) were also calculated. Percent change was calculated using the following equation: $\frac{{{\text{Value}}\,\,{\text{after}} - {\text{Value}}\,{\text{before}}}}{{{\text{Value}}\,{\text{before}}}} \times \,100$ Comparison of study variables between the two groups at each point of time was done using T-test when the variable was normally distributed and Mann–Whitney U test when the variable was not normally distributed using Monte Carlo corrected significance levels. Comparing different time points in each group separately was done using repeated measures ANOVA for normally distributed variables and Friedman test for non-normally distributed variables, and both were followed with multiple pairwise comparisons using Bonferroni adjustment. All tests were 2-tailed [35]. Significance was set at *p* value < 0.05. Data were analyzed using IBM SPSS statistical software version 23.0.

## Results

### Results of demographic and OLP lesion's characteristics

In this clinical trial, 20 patients (14 females and six males) were assessed for eligibility criteria from January 2019 to April 2020. All enrolled patients completed the study. Ten patients in each group were included with 6 (60%) and 8 (80%) females in the test and control groups respectively. Mean ± SD age was 51.50 ± 8.00 in the lycopene group and 45.90 ± 9.63 in the corticosteroids group. Comparisons revealed no significant difference between both groups as regards sex (*p* = 0.63) and age (*p* = 0.11).

All patients suffered from clinically and histopathologically diagnosed EOLP involving 56 oral sites. The most common site of involvement was the buccal mucosa (57.14%), followed by the tongue and gingiva (17.85% each), the palate and the lips (3.57% each).

### Results of clinical outcomes

In both studied groups, there was a statistically significant reduction (*p* < 0.001*) in the mean score values of pain, lesion severity and activity at the end of treatment (week 8). Pairwise comparisons of the clinical outcomes at all assessment times are shown in Tables 1 and 2.

**Table 1:** Changes in Numeric rating scale (NRS) in both study groups at all assessment times

NRS	Lycopene group (n = 10)	Corticosteroids group (n = 10)	T-test *p* value
	Mean ± SD		
Baseline	5.90 ± 1.85^a^	5.60 ± 1.43^a^	*p* = 0.69
4 weeks	3.30 ± 1.42^b^	2.80 ± 1.23^b^	*p* = 0.41
8 weeks	2.10 ± 1.20^b^	3.00 ± 1.56^b^	*p* = 0.17
Repeated measures ANOVA *p* value	*p* < 0.001*	*p* < 0.001*	

^a,b^Different superscripted letters denote statistically significant differences between different time points in each group using Bonferroni adjustment for multiple pairwise comparisons

SD, standard deviation; n, number of patients

*Statistically significant at *p* value < 0.05

**Table 2:** Mean ± Standard deviation differences of Escudier et al. scores throughout the study period

	Site score		Severity score		Activity score	
	Lycopene group (n = 10)	Corticosteroids group (n = 10)	Lycopene group (n = 10)	Corticosteroids group (n = 10)	Lycopene group (n = 10)	Corticosteroids group (n = 10)
Baseline	4.50 ± 2.12^a,b^	3.40 ± 1.58^a^	4.70 ± 3.09^a^	5.90 ± 2.23^a^	7.70 ± 4.27^a^	8.20 ± 3.94^a^
Week 4	3.50 ± 1.78^a,b^	2.10 ± 1.00^b^	3.30 ± 2.91^a,b^	3.10 ± 2.51^b^	4.40 ± 3.37^a,b^	3.10 ± 2.b^b^
Week 8	3.30 ± 1.83^a,b^	1.90 ± 0.88^b^	1.70 ± 1.95^b^	2.30 ± 1.70^b^	2.00 ± 2.26^b^	2.30 ± 1.70^b^
Friedman test *p* value	*p* < 0.001*	*p* < 0.001*	*p* < 0.001*	*p* < 0.001*	*p* < 0.001*	*p* < 0.001*

^a,^^b^Different superscripted letters denote statistically significant differences between different assessment times in each group using Bonferroni adjustment

n, number of patients

*Statistically significant at *p* value < 0.05

After eight weeks, 60% of patients in each of the test and control groups experienced almost complete resolution of signs and symptoms. The other 40% experienced scores improvement with some residual lesions and pain. Patients taking lycopene showed no adverse side effects throughout the study period. On the other hand, about 50% of patients taking corticosteroids showed adverse side effects as facial puffiness, gastrointestinal disturbances, and general weakness.

Inter-group comparisons revealed no statistical significant difference in the clinical outcomes at baseline, weeks 4 and 8 as shown in Table 1 and Additional file 1: Table S1: Median, Inter Quartile Range (IQR) and inter-group comparisons of lesion scores at all assessment times.

### Results of biochemical outcomes

Biochemical analysis showed a statistically significant decrease in serum 8-isoprostane levels after 8 weeks of lycopene administration (*p* = 0.01*). Inter-group comparisons revealed that concentrations of 8-isoprostane at the same assessment time were significantly lower in the lycopene than the corticosteroids group (*p* < 0.001*) as shown in Table 3.

**Table 3:** Serum levels of 8-isoprostane in both study groups at baseline and after 8 weeks

8-isoprostane serum levels	Lycopene group (n = 10)	Corticosteroids group (n = 10)	Mann–Whitney U *p* value
	Mean ± SD Median (IQR)		
Baseline	96.21 ± 35.06 101.90 (60.80, 114.68)	108.73 ± 46.11 95.35 (62.05, 162.00)	*p* = 0.63
Week 8	59.94 ± 4.04 58.35 (57.65, 60.48)	93.65 ± 38.74 78.90 (62.40, 120.80)	*p* < 0.001*
Paired t-test *p* value	*p* = 0.01*	*p* = 0.45	

SD, standard deviation; IQR, inter quartile range; *n* number of patients

*Statistically significant at *p* value < 0.05

## Discussion

Lichen planus is an autoimmune disease with debatable pathogenesis [5]. Oral Lichen Planus is considered a serious variant that has long been discussed as an added burden to affected patients [1, 36]. Conventional treatments of OLP are often inconvenient, the thing that has presented different modalities, mainly antioxidants, as alternative therapies in such situations [13].

In this trial, the test group received 10 mg of lycopene for eight consecutive weeks. In OLP, previous clinical trials tested lycopene as a therapeutic agent [7, 37–40]. The length of treatment followed in the present study was based on the trials by Saawarn et al. [37], Shekhawat et al. [38] and Kushwaha et al. [7] that used LYC for eight weeks. The dose was inconsistent with the mentioned trials due to the different study designs and constituents of the medication used. Besides, the following trial aimed to use the least effective dose of pure lycopene as a single treatment. Therefore, the selected dosage (10 mg) was based on a review by Kaur et al. [28], which stated that among all clinical trials that used moderate amounts of LYC, the dose rarely exceeded 10 mg. This was also in accordance with Hazzaa et al. [40] who recently used the same dose for OLP treatment.

The control group received Prednisolone for eight consecutive weeks as an initial constant dose (40 mg) followed by tapered doses. Overall treatment duration, initial constant dose, and the amount of incremental reduction followed were all based on the clinical trial by Kushwaha et al. [7]. As regards the duration of the initial dose, there are no specific guidelines. Kini et al. [29] stated in their review that systemic corticosteroid regimens in OLP vary according to patient-related factors as weight, medical status, the severity of oral lesions, and previous response to treatments proposed. Moreover, a starting dose of 50 mg in OLP patients was reported with variable durations not exceeding two months [41]. In this trial, all enrolled patients were recalcitrant to previous topical steroids therapy. Owing to their need to benefit from Prednisolone as an alternative with the least possible side effects, treatment was started with 40 mg for four consecutive weeks.

Demographic and OLP lesions' characteristics were in accordance with its nature as have been studied for so long. It has a female predilection affecting an age range of 30 to 60 years, and lesions affect mainly the buccal mucosa, followed by the tongue and other oral sites [19, 42].

**Fig. 1:**
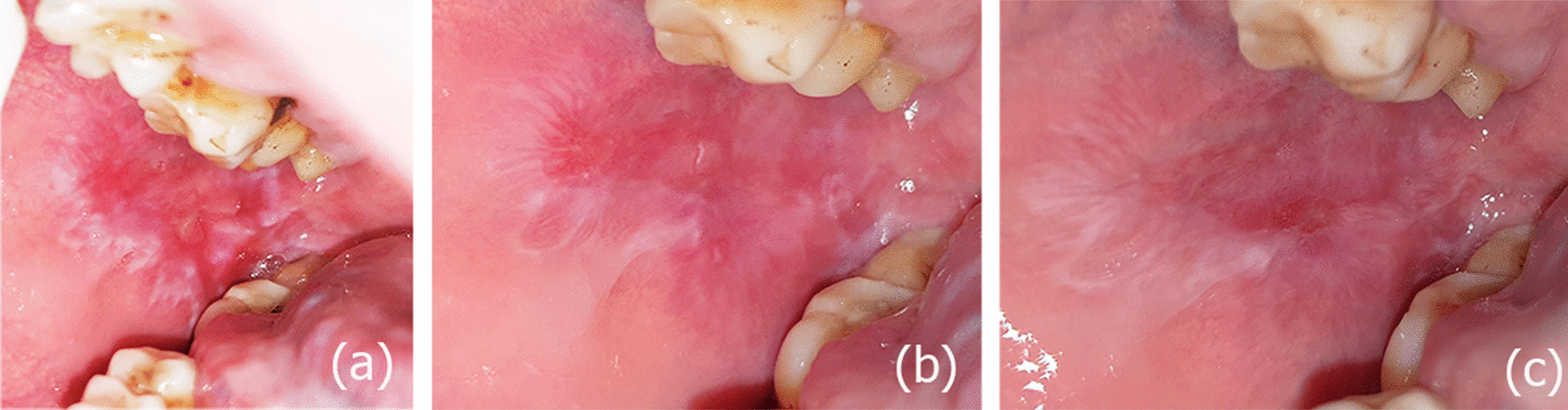
OLP lesions changes along all assessment times in a case of the lycopene group (**a**–**c**). **a** Baseline assessment revealing erosive and white lesions of the right buccal mucosa. **b** 4weeks follow up showing improvement of the degree of redness and reduction of striations. **c** 8 weeks follow up showing almost complete resolution of EOLP

**Fig. 2:**
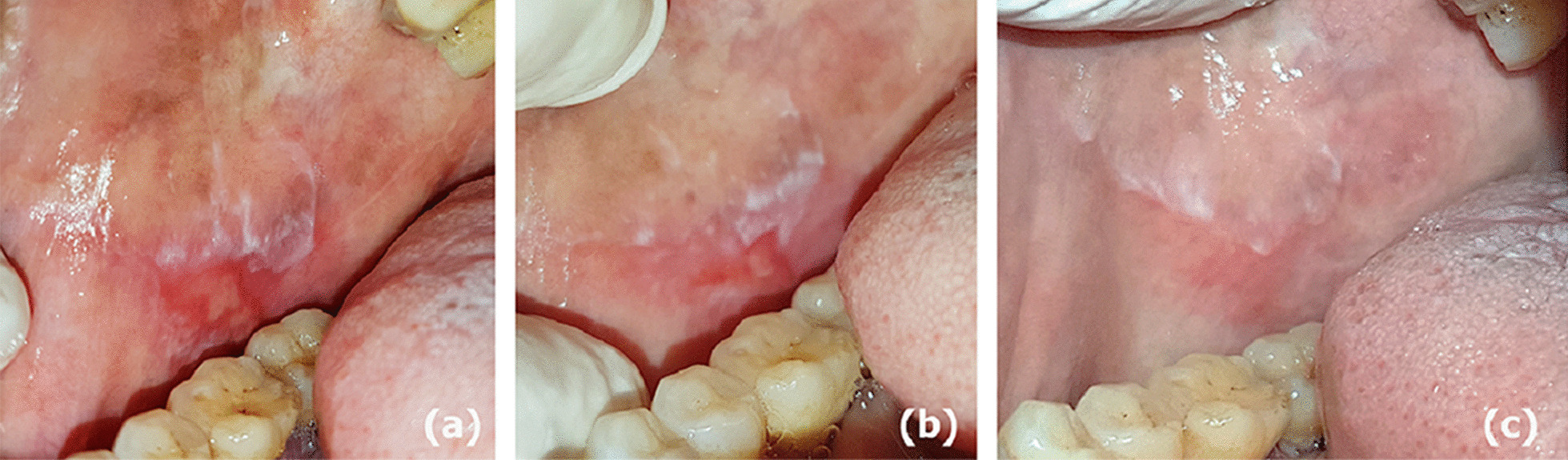
OLP lesions changes along all assessment times in a case of the corticosteroids group (**a**–**c**). **a** Baseline assessment revealing erosive and white lesions of the right buccal mucosa. **b** 4 weeks follow up showing reduction of lesion size. **c** 8 weeks follow up showing almost complete resolution of EOLP

At the end of treatment (week 8), lycopene and Prednisolone were found to significantly reduce the signs and symptoms of OLP. Their effects on the lesions at that time were manifested as reduction of the size of red lesions, regression of their severity (degree of redness), and conversion of erosions and ulcers to erythema or almost their complete disappearance (Figs. 1, 2). White lesions showed either reduction or changes in the pattern of striations in some patients. Those results are parallel with Kushwaha et al. [7] who noted a significant decrease in burning sensation and lesion severity in both lycopene and prednisolone groups after eight weeks of treatment. In addition, Saawarn et al. [37] and Shekhawat et al. [38] found that giving LYC for the same duration has significantly decreased signs and symptoms as compared to placebo and levamisole respectively. However, authors of the three trials used different doses (4 mg and 8 mg) of LYC in combination with multiple antioxidants which might have acted synergistically with it providing the positive effects described. Moreover, Hazzaa et al. [40] revealed marked reduction in OLP manifestations after using the followed dose by the present trial (10 mg) for 8 weeks.

The positive results offered by lycopene administration can be explained by its wide range of beneficial properties. This potent carotenoid antioxidant is famous for its quenching ability that efficiently targets singlet oxygen [16]. Interestingly, this potency was found to be twice as high as that of its isomer (beta carotene) and ten times higher than that of alpha-tocopherol [43]. As singlet oxygen interacts with cellular macromolecules, lycopene is considered influential in protecting cells from oxidative injury [44]. In addition, LYC downregulates the synthesis and release of a range of pro-inflammatory markers (TNFα, IL4, IL6, IL8, IL1β) [45, 46], this not only inhibits inflammation but also minimizes the rate of production of ROS and free radicals by breaking off the promoting action of those mediators on their release [47]. However, these effects need rigorous demonstration in OLP at higher molecular levels.

The control group's results can be attributed to the mechanisms of action of corticosteroids as potent anti-inflammatory and immunosuppressive agents. They maintain cell membrane integrity, inhibit phagocytosis, and lysozyme release, and additionally, they can suppress T cell functions; therefore, downregulating the cell-mediated immunity [48].

The improvement in clinical signs and symptoms without complete resolution that some patients experienced in both study groups may be because OLP is a dynamic disease process with constantly fluctuating manifestations. In general, the not fully explained etiology renders various treatment modalities serve as symptom relievers rather than actual curative remedies [49, 50].

Throughout the treatment phase, there was no significant difference in OLP subjective and objective changes between both proposed interventions. On the contrary, Kushwaha et al. [7] reported significantly lower pain scores after 8 weeks in the prednisolone rather than the LYC group. This disparity could be due to the differences in doses and compositions of used LYC, as well as the design that both studies followed. The higher dose (10 mg) of pure LYC might have enhanced its promising roles in OLP to reach an overall close clinical performance to that of oral steroids.

Amirchaghmaghi et al. [11] noted high levels of oxidative stress in patients with OLP reflected by elevated 8-isoPGF2α plasma levels as compared to healthy controls. Up to the present, no study evaluated the effect of treatment on the levels of this biomarker in such a disease. In this clinical trial, lycopene was found to significantly reduce 8-isoprostane levels at the end of treatment (week 8), and as compared to Prednisolone. Being the first trial to investigate such an effect, it was difficult to correlate the present findings with studies having the same design. Even though, the results were in accordance with Visioli et al. [51] who found a significant decrease in urinary 8-isoPGF2α levels after three weeks of different tomato products consumption, providing about 8 mg of lycopene per day. Moreover, 10 mg of LYC was capable of lowering the mean expression levels of salivary malondialdehyde in patients with OLP [40].

It is worth mentioning that although 8-isoprostane is well known to be released from non-enzymatic free radical catalyzed reactions, some investigators found that it may be produced from an alternative enzymatic mechanism depending on cyclooxygenase enzymes (COX1 & COX2). Nonetheless, studies that highlighted the importance of specifying its exact release mechanism in accordance to individual studies revealed the domination of the chemical (free radical dependant) mechanism in humans [12, 52].

According to the present results, Prednisolone didn't affect 8-isoPGF2α levels significantly regardless of the previously reported inhibitory effect of both studied therapeutic modalities on the COX2 enzyme [53, 54]. This is coordinate with Montuschi et al. [55] who noted that neither selective nor non-selective cyclooxygenase inhibitors affected exhaled 8-isoPGF2α levels in chronic obstructive pulmonary disease (COPD) patients. Based on those findings, it could be suggested that the marked reduction in 8-isoprostane concentration after lycopene administration is a reflection of decreased lipid peroxidation as a result of lowered oxidative stress. However, assessment of the effects of both medications on COX in patients of the present study would have given more reliable conclusions about the release mechanism of 8-isoprostane.

Lycopene has proven optimistic results in the treatment of resistant EOLP based on the grounds of this research. However, it is recommended to evaluate the outcomes of other forms, doses and regimens of LYC through studies of different designs and larger sample size. Furthermore, this trial advocates oxidative stress assessment over longer durations to achieve more comprehensive conclusions about the stability offered by LYC as a therapeutic modality for OLP. Moreover, there is a need for larger evidence about the status of 8-isoprostane and its dominating release mechanism in OLP through upcoming research. In addition, conducting a blinded approach would avoid any possible chance of bias that might have not been avoided in the present trial where blinding of the operators and patients was difficult due to the different formulations and regimens of the used medications.

## Conclusions

Lycopene and prednisolone have promising therapeutic effects on patients with recalcitrant erosive oral lichen planus. As lycopene has the ability to reduce oxidative stress and showed no adverse side effects, it could be useful in OLP treatment. 8-isoprostane is a reliable biomarker of lipid peroxidation that can be significantly reduced by lycopene.

## Supplementary Information

**Additional file 1.** **Table S1:** Median, Inter Quartile Range (IQR) and inter-group comparisons of lesion scores at all assessment times.

## Data Availability

The datasets generated during and/or analysed during the current study are available from the corresponding author on reasonable request.

## Abbreviations

LP: Lichen planus
OLP: Oral lichen planus
EOLP: Erosive oral lichen planus
ROS: Reactive oxygen species
8-iso-PGF2α: 8-Isoprostane
LYC: Lycopene
NRS: Numeric rating scale
OD: Optical density
COX: Cyclooxygenase
COPD: Chronic obstructive pulmonary disease
