# Supplementary material for: Serum 8-isoprostane levels in patients with resistant oral lichen planus before and after treatment with lycopene: a randomized clinical trial
Source: BMC Oral Health. 2021 Jul 15;21:343. doi: 10.1186/s12903-021-01711-z (PMC8281687; doi:10.1186/s12903-021-01711-z)
Supplement: Supplementary file 1 — Additional file 1. Table S1: Median, Inter Quartile Range (IQR) and inter-group comparisons of lesion scores at all assessment times. [file 12903_2021_1711_MOESM1_ESM.pdf]

Serum 8-isoprostane levels in patients with resistant oral lichen planus before and after treatment with lycopene: A Randomized Clinical Trial

Aliaa Abdelmoniem Bedeir Eita*, Azza Mohamed Zaki,
Sabah Abdelhady Mahmoud

Supplementary Table S1: Median, Inter Quartile Range (IQR) and inter-group comparisons of Escudier et al. lesion scores at all assessment times

Site score	Lycopene group (n=10)	Corticosteroids group (n=10)	Mann Whitney U p value
	Median (IQR)		
**Baseline**	4.00 (2.75, 6.00)	3.00 (2.75, 4.50)	P = 0.25
**4 weeks**	4.00 (1.75, 4.50)	2.00 (1.00, 2.50)	P = 0.09
**8 weeks**	3.50 (1.75, 4.50)	2.00 (1.00, 2.00)	P = 0.09
**Severity score**	**Median (IQR)**		**Mann Whitney U p value**
**Baseline**	4.00 (3.00, 5.00)	5.90 (4.50, 6.75)	P = 0.08
**4 weeks**	2.50 (1.00, 5.00)	2.00 (1.00, 4.75)	P = 0.91
**8 weeks**	1.00 (0.00, 2.50)	2.00 (1.00, 3.25)	P = 0.28
**Activity score**	**Median (IQR)**		**Mann Whitney U p value**
**Baseline**	6.50 (5.00, 8.25)	8.00 (5.00, 10.00)	P = 0.63
**4 weeks**	4.50 (1.00, 7.25)	2.00 (1.00, 4.75)	P = 0.53
**8 weeks**	1.50 (0.00, 3.00)	2.00 (1.00, 3.25)	P = 0.44

*statistically significant at p value < 0.05

n: number of patients
